# MyoNet: A Transfer-Learning-Based LRCN for Lower Limb Movement Recognition and Knee Joint Angle Prediction for Remote Monitoring of Rehabilitation Progress From sEMG

**DOI:** 10.1109/JTEHM.2020.2972523

**Published:** 2020-02-13

**Authors:** Arvind Gautam, Madhuri Panwar, Dwaipayan Biswas, Amit Acharyya

**Affiliations:** 1 Department of Electrical EngineeringIndian Institute of Technology Hyderabad 233600 Hyderabad502205India; 2 imec 482183 3001LeuvenBelgium

**Keywords:** sEMG, movement classification, joint angle prediction, signal processing, LSTM, CNN, transfer learning

## Abstract

The clinical assessment technology such as remote monitoring of rehabilitation progress for lower limb related ailments rely on the automatic evaluation of movement performed along with an estimation of joint angle information. In this paper, we introduce a transfer-learning based Long-term Recurrent Convolution Network (LRCN) named as ‘*MyoNet*’ for the classification of lower limb movements, along with the prediction of the corresponding knee joint angle. The model consists of three blocks- (i) feature extractor block, (ii) joint angle prediction block, and (iii) movement classification block. Initially, the model is end-to-end trained for knee joint angle prediction followed by transferring the knowledge of a trained model to the movement classification through transfer-learning approach making a memory and computationally efficient design. The proposed *MyoNet* was evaluated on publicly available University of California (UC) Irvine machine learning repository dataset of the lower limb for 11 healthy subjects and 11 subjects with knee pathology for three movements type-walking, standing with knee flexion movements and sitting with knee extension movements. The average mean absolute error (MAE) resulted in the prediction of joint angle for healthy subjects and subjects with knee pathology are 8.1 % and 9.2 % respectively. Subsequently, an average classification accuracy of 98.1 % and 92.4 % were achieved for healthy subjects and subjects with knee pathology, respectively. Interestingly, the significance of this study in itself is promising with substantial improvement in the performance compared to state-of-the-art methodologies. The clinical significance of such surface electromyography signals (sEMG) based movement recognition and prediction of corresponding joint angle system could be beneficial for remote monitoring of rehabilitation progress by the physiotherapist using wearables.

## Graphical abstract

A transfer-learning based Long-term Recurrent Convolution Network (LRCN) deep learning framework named MyoNet is proposed for lower limb movement recognition along with prediction of corresponding knee joint angle from sEMG signal recording for remote monitoring of rehabilitation progress.

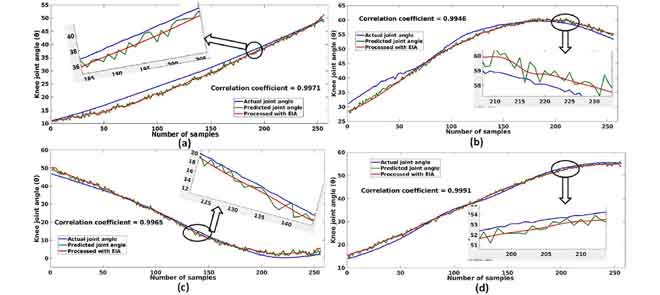

## I. Introduction

NEE injuries due to sports or accidents like anterior cruciate ligaments (ACL) injury, meniscus injury, and sciatic nerve injury, and knee osteoarthritis are the most common causes of disability to the person of any age globally [1]–[4]. The assistive technology including monitoring the rehabilitation progress has significant potential in improving the quality of life of such differently-abled persons [4]–[8]. For diagnosing neuromuscular and skeletal disorder, the diagnostic devices use gait analysis for classification and assessment of lower limb motion [9]. After diagnosing, patients required therapeutic exercises for the purpose of rehabilitation in training the muscles associated with activity of daily life (ADL) prescribed by the physiotherapist [6], [7], [10]. However, traditional clinical rehabilitation techniques require extensive gait laboratory setting which is a long, inconvenient, expensive and sometimes unavailable in remote areas [9], [11], [12]. These limitations necessitate a remote monitoring of the rehabilitation progress using wearables, which not only control the assistive devices such as exoskeleton but can also provide the recovery feedback to the user as well as give assistance to the clinicians in the assessment and treatment of the patients [6], [8], [11], [12].

The remote monitoring of rehabilitation progress especially for lower-limb related ailments, needs more efficient and intelligent framework to decode user’s intention along with joint information in performing particular movement [8], [13]. The working principle for such framework includes two important tasks, viz. (i) decoding user intention and (ii) prediction of joint angle information. The first task is ‘decoding user intention’, which is usually accomplished by applying pattern recognition techniques on surface electromyography signals (sEMG) captured from user’s muscles for inferring the intent of performing desired movements [14]–[16]. However, since the lower limb muscles are present deep beneath the skin with significant overlap among them, therefore the classification of sEMG signals from such muscles is more challenging when compared to the upper limb muscles [14]. Due to this very reason, many researchers have introduced a combination of classical signal processing and pattern recognition techniques in the classification of lower limb movements from sEMG signals. Naik et al. [14] applied independent component analysis via entropy bound minimization (ICA-EBM) with linear discriminant analysis (LDA) classifier for recognizing of lower limb movements of persons with and without knee pathology. Varol *et al.* [13] proposed a multiclass real-time intent recognition based on sEMG signals approach for powered lower limb prosthesis. In another study, Joshi et al. [17] used Bayesian information criterion and linear discriminant analysis for classification of different gait phases of the lower limb.

On the other hand, the second task of remote monitoring system for rehabilitation, as mentioned above is ‘prediction of joint angle information’ that can provide accurate assistive torque to the user [6]. In this context, Kianifar *et al.* [2] used inertial sensor for estimation of joint angle for automatic assessment of dynamic knee valgus and risk of knee injury during the single leg squat. Zhang *et al.* [8] introduced an autoregressive integrated (ARI) model to predict time series knee joint angle for network based rehabilitation system. Chen *et al.* [18] proposed artificial neural network for estimation of knee joint angle using EMG signals and functional electrical stimulation (FES) parameters such positive pulse amplitude, positive pulse width and negative pulse width. Lui *et al.* [19] used time-domain features from sEMG for the prediction of knee joint angle based on generalized regression neural network. Recently, Huang *et al.* [20] used recurrent neural network for prediction of real-time intended knee joint motion from combination of sEMG and inertial data.

Based on the aforementioned observations, it can be noted that the state-of-the-art methodologies used a combination of sEMG signal with inertial sensor data and goniometers for joint angle prediction [2], [6], [18]–[20], whereas for movement classification only sEMG signal is being preferred. Therefore, to eliminate the additional computational cost incurred by other signals processing in predicting joint angle, in this study, we focused on executing the movement classification and joint angle prediction on a single unified platform using sEMG signal only, removing the need of inertial sensor data and goniometer data. Additionally, the state-of-the-art methodologies for sEMG based lower limb movement classification as well as joint angle prediction, suffer from accuracy issues due to handcrafted feature selection and extraction before classifying the intended movements and predicting the joint angle. Furthermore, the feature selection and extraction put a significant amount of time due to the human intervention and exertions in finding suitable features, whereas feature extraction increases computational time and complexity. Thus optimal, accurate, and fast feature selection as well as extraction for classification of movement and estimation of joint angle are still considered to be a challenging task for the envisaged remote monitoring of rehabilitation progress device using wearables.

Therefore, motivated by aforementioned arguments and observations, in this paper, we propose a transfer-learning based deep learning framework ‘*MyoNet*’ on a single unified platform for accurate classification of lower limb movements along with the prediction of corresponding knee joint angle from four channel sEMG recordings. The proposed framework is designed by utilizing the Long-term Recurrent Convolution Network (LRCN), exploiting the data-driven feature engineering property and precluding handcrafted feature extraction and selection procedure [11], [12], [21]–[23]. Further, the LRCN delivers end to-end learning from the raw data in data-driven fashion, which enables high level features extraction, helping in understanding, determining and distinguishing the hidden information from the data regarding their clinical patterns due to its hierarchical structure. Thereby, it eliminates the need of domain expertise thus, improves performance. Further, Transfer-Learning is applied to make a memory and a computationally efficient design. This is done by transferring the knowledge of a model trained for prediction of joint angle to the model of classification of movements. The novelty of this work lies in developing an accurate, hybrid deep learning model having the capability to recognize the lower-limb movements, and predict joint angle information of the performed limb movement which is essential especially for remote monitoring of rehabilitation progress where expert clinicians and physiotherapist are not present. The proposed *MyoNet* was successfully evaluated on the publicly available UC Irvine Machine Learning Repository dataset, comprising of 11 healthy subjects and 11 subjects with knee injuries for three movement classes i.e., walking, sitting, and standing [24]. We achieved an average accuracy of 98.1 % and 92.4 % for classifying the limb movements of healthy and knee-pathology subjects along with their joint angle having 8.1 % and 9.2 % mean absolute error, respectively. The successful classification of lower limb movement along with the prediction of joint angle information demonstrates the usefulness of LRCN model in accessing the rehabilitation conditions particularly for subjects with knee pathology where joint angle information in very important in evaluating the overall conditions in remote monitoring settings.

The remainder of the paper is structured as follows- Section II provides motivational background and method for data acquisition system, Section III discuss the proposed MyoNet framework along with training and evaluation procedure, Section IV presents the results and discussion and Section V concludes the paper.

## II. Motivational Background and Method

### A. Long-Term Recurrent Convolutional Network (LRCN)

**FIGURE 1.**
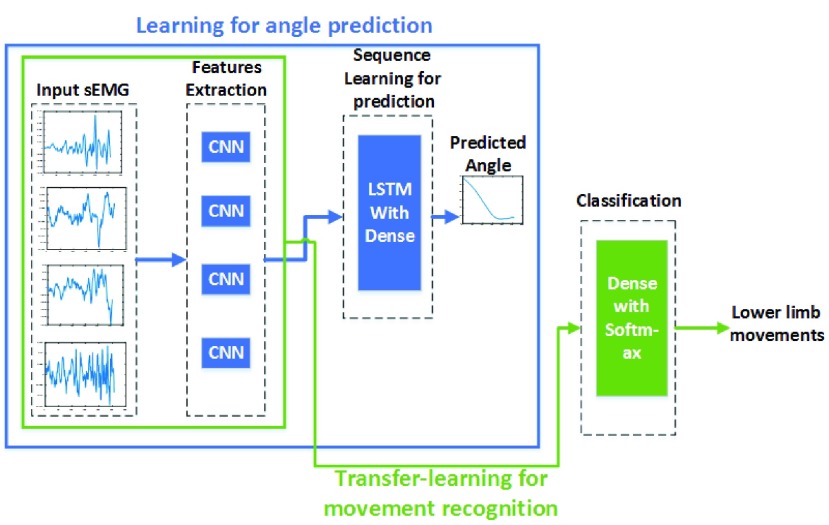
Transfer-learning based proposed LRCN architecture of DNN.

The proposed transfer-learning based LRCN architecture of deep neural network (DNN), shown in Fig. 1 consists of Convolutional Neural Network (CNN) for feature extraction from input data followed by LSTM with a dense layer for joint angle prediction and another dense layer with softmax loss function at the output for interpreting the features across time step for classification. For movement’s classification, transfer-learning technique is applied wherein learned knowledge of sEMG data during the angle prediction is directly transferred to the dense layer of movement classification. Thus, eliminates the need of extracting the sEMG features which already being learned during the angle prediction task. Thereby, it provides saving in the computational cost and time along with the memory requirement, owing to the fact that it shares the knowledge of angle prediction tasks.

The CNN extracts the discriminant features in a data-driven fashion from raw input data while training. The CNNs are described by a set of convolutional filters (consists of weights which slides over input) gives outputs feature maps, followed by the activation function, pooling layer, and fully connected layer which realizes the classification [25]. Whereas, LSTM units are a type of recurrent neural network (RNN) [26] which enables long-range learning. Typically, LSTM units contain a hidden state activated with nonlinear function, which uses a learned gating function to allow that state to propagate without modification, be updated, or be reset. Recently, LSTMs have achieved impressive results in biomedical applications [21], speech recognition [27], and language translation [28], [29] and computer-vision applications [30]. In a recent study, Biswas *et al.* [21] proposed a CorNET network for PPG based heart rate estimation and biometric identification. Motivated by the progress and achievement of LRCN models, in this paper our attempt is to propose an LCRN architecture based on sEMG signal for classification of lower limb movements along with prediction of their knee joint angle.

### B. Method for Data Acquisition System

In this study, 4 channels sEMG and 1 channel goniometer data were considered from lower limb of 22 male participants older than 18 year of age, publicly available at UC Irvine Machine Learning Repository [24]. The participants are 11 healthy and 11 with knee pathology (i.e. six with anterior cruciate ligaments (ACL) injury, four with meniscus injury, and one has sciatic nerve injury). For sEMG and knee joint angle data collection, Datalog MWX8 by Biometrics Ltd. and SG150B goniometer is used.

The four channels sEMG electrodes were placed 20 mm apart with a high input impedance greater than 10 M ohm to allow sampling without conducting gel. The sampling frequency for data collection is 1000 Hz with 14 bit resolution and data samples were bandpass filtered in frequency range between 20 Hz to 460 Hz. The four channels of sEMG electrodes were placed on the surface of muscles which are (i) vastus medialis (vm), (ii) semitendinosus (st), (iii) biceps femoris (bf), and (iv) rectus femoris (rf) and the goniometer was kept to the external side of knee joint. The left leg and affected limb of the individuals were selected for the normal subjects and subjects with knee pathology respectively.

The sEMG data and knee joint angle were recorded for three physical activities of the lower limb such as walking on level ground (gait), sitting with knee extension movements and standing with knee flexion movements respectively. These three movements were chosen because they do not require extra weights, dumbbell and fitness equipment in doing activity of daily life (ADL) and rehabilitation process. It can be noted that the dataset does not include the sEMG and knee joint angle data corresponding to the transition phase i.e. sitting to standing and walking to sitting etc.

## III. Proposed MyoNet Model

**FIGURE 2.**
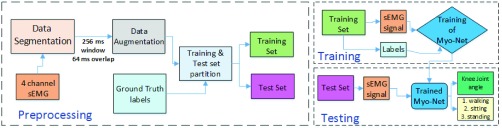
Workflow of the proposed *MyoNet* framework.

The proposed *MyoNet* model is designed for the classification of three lower limb movements along with the prediction of corresponding knee joint angle, which includes walking, sitting with knee extension movement, and standing with knee flexion movement. Fig. 2 shows the workflow of the proposed *MyoNet* model comprising of preprocessing, model development and training-testing steps.

### A. Preprocessing

The preprocessing step consists of data segmentation, data augmentation, 3-fold training and test set partition along with annotation with ground truth, and data normalization to zero mean and unit variance. All the four channels of sEMG data were considered for input to design the customized deep learning model based on the literature. The four channel input sEMG data are then sectioned into 256 ms window with 64 ms overlap similar to the study reported by Naik *et al.* [14] to ensure the delay is within the tolerable limit i.e. 300 ms window so that natural dexterity can be achieved in real-time [31], [32]. Further, it can be noted that the performance of the model increases with increase in window length as reported in [32].

Since deep learning algorithms demand a huge amount of data for training and testing the model for better generalization capability and performance analysis in real-time. However, collecting repetitive activity data for long-duration from individuals with knee pathologies is challenging. Therefore, data augmentation is a favored technique in the field of deep learning for building a robust and generalized solution for small datasets [33]. The recent study [34], revealed the importance of data augmentation wherein virtual data were generated by mixing Gaussian noise on sEMG data which shown increase in the performance of deep learning model. The performance increment is achieved due to inclusion of those data samples in training which may occur in real-time. Thus, in this study Gaussian noise is added to the segmented raw sEMG data, which generate diverse possible variations in the input data resulting in 10x larger dataset size. Equation (1) expresses the data augmentation technique adopted from [11], where Dv is virtual data, Do is the original input data and wgn is a weighting parameter generating an $m$-by-$n$ matrix of white Gaussian noise samples and $p$ is the power. The power p of the white Gaussian noise is chosen such that the signal to noise ratio of the augmented data equals to 25 based on the study conducted by [34].

\begin{equation*} D_{v}=D_{o}+wgn(m,n,p)\tag{1}\end{equation*}

Based on the previous study by [14] on the same dataset, we have employed a similar approach for partitioning the data into a training set and test set for a fair comparison. The approach used is a simple holdout method, i.e. k-fold cross-validation strategy for k equal to three in the vision of improving the consistency for the prediction and classification results. Thus, the datasets are arbitrarily divided into k subsets, and the method is repeated for k times resulting k-folds. In every fold, one of the k subsets is considered as test dataset, while the k-1 subset is treated as the training dataset. The mean absolute error and mean classification accuracy across all the k-folds is reported in the results. Furthermore, sEMG data is normalized to zero mean and unit variance based on training dataset for faster conversion of gradient descent and prevention of overfitting problem [35].

### B. Network Architecture of the Proposed MyoNet

**FIGURE 3.**
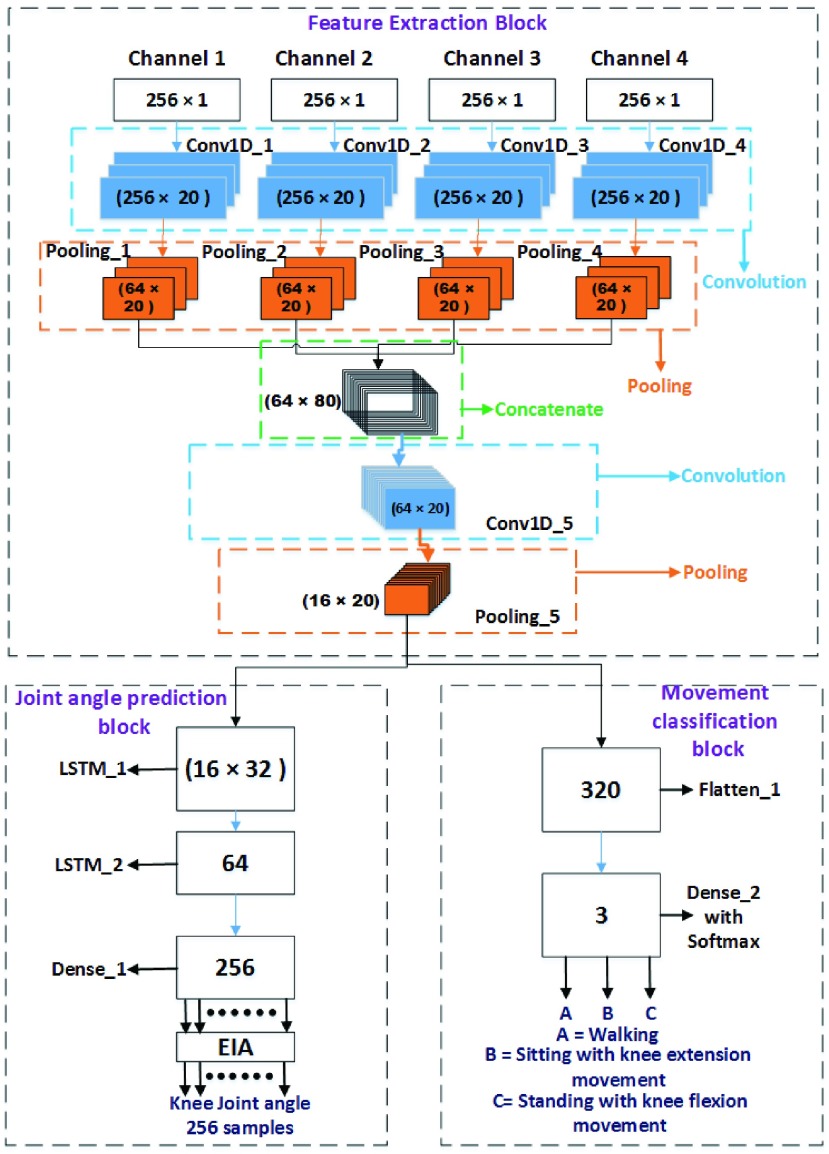
Network architecture of proposed *MyoNet* model.

Fig. 3 represents the network architecture of the proposed *MyoNet* model which comprises of feature extractor, joint angle predictor, and movement classification blocks. For input, all the four channels of sEMG were considered in this study, therefore, the first stage consists of four convolutional layers, working in parallel with Relu activation function followed by max-pooling and dropout layers to extract all the features simultaneously from four channels. Further, all the obtained feature-maps are concatenated before inputting to the second stage. The second stage consists of only one convolutional layer with Relu activation function followed by max-pooling and dropout layer. Then, the output of the feature extractor block is fed concurrently to both joint angle predictor block and movement classification block for further processing. The joint angle predictor block is having two layers of LSTM units with linear activation function in series followed by a dense layer for the prediction of knee joint angle based on features extracted from sEMG signals. Likewise, the classification block consists of flatten layer followed by dense layer with softmax loss function, which helps in the classification of movements based on the extracted features.

The key factor which affects the performance of the DNN model is the selection of the optimally tune hyper-parameters. These parameters can be categorized into two types such as architectural and training parameters. For the selection of architectural parameters, we have considered the number of layers, number of filters/kernels in each layer, size of each filter, stride rate and pooling size. While, for training parameters we include learning rate, type of back propagation algorithm, activation function, loss function, dropout and number of epochs, etc. The proposed *MyoNet* model utilizes 20 filters of size $11\times 1$, stride rate of 1, and $4\times 1$ max-pooling in all the convolutional in the feature extractor block. For joint angle predictor block, a size of 32 and 64 memory units were used for first and second LSTM layer respectively along with 16640 neurons in the dense layer linked to 256 outputs. Whereas, in movement classification block, 963 neurons linked to three outputs in the dense layer were used. For model training, batch size of 25, 70 epochs, dropout with 50 % probability level, and Adam optimizer with default learning rate of 0.001 were set. These architectural details of *MyoNet* is provided in Table 1. The obtained hyper-parameters were optimized using heuristic grid search, which yielded superior performance compared to state-of-the-art methodologies.

**TABLE 1:** MyoNet Model Parameters Detail

Block			MyoNetModel	
			Output shape	Weights + biases
Feature extraction	Stage 1	Input_1	256, 1	0
		Input_2	256, 1	0
		Input_3	256, 1	0
		Input_4	256, 1	0
		Conv1D_1	256, 20	240
		Conv1D_2	256, 20	240
		Conv1D_3	256, 20	240
		Conv1D_4	256, 20	240
		Pooling_1	64, 20	0
		Pooling_2	64, 20	0
		Pooling_3	64, 20	0
		Pooling_4	64, 20	0
	Concatenate		64, 80	0
	Stage 2	Conv1D_5	64, 20	17620
		Pooling_5	16, 20	0
Joint angle prediction	LSTM_1		16, 32	6784
	LSTM_2		64	24832
	Dense_1		256	16640
Movement classification	Flatten_1		320	0
	Dense_2		3	963

### C. Processing of Predicted Joint Angle From Empirical Iterative Algorithm

**FIGURE 4.**
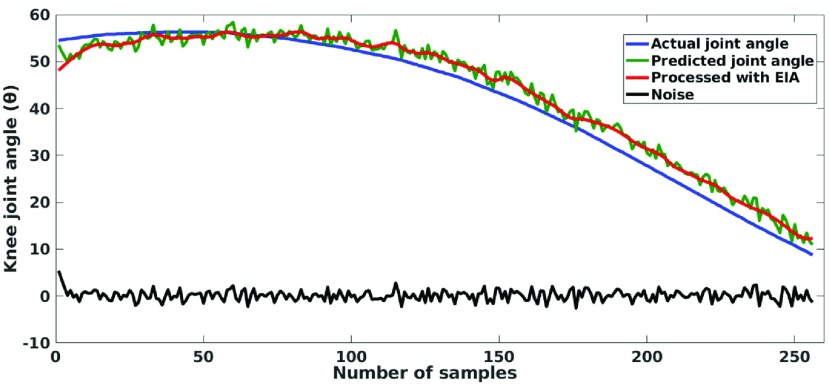
Comparison of actual, predicted and EIA processed joint angle with noise.

The prediction of knee joint angle from sEMG signals captured from lower limb muscles is challenging because the former signals are complex in nature and associated with crosstalk issue due to physiology of the related and other muscles placements. Thus, in our attempt from sEMG to knee joint angle prediction by exploiting the data-driven deep learning approach using CNN and LSTM showed interesting results as depicted in Fig. 4. However, it can be noticed from Fig. 4 that the proposed model is able to accurately predict the trend of increasing or decreasing joint angle but with some embedded unwanted noise which is due the deviation of predicted joint angle from the actual joint angle. Thus, to remove the unwanted noise, we have used our previously published work i.e. a data-driven iterative low pass filtering ‘Empirical Iterative algorithm’ (EIA) [36], inspired by Empirical Mode Decomposition (EMD) [37] to smoothen the predicted knee joint angle. The working principle of EIA is to get the trend of a signal by finding mid-point of consecutive local maxima and minima and vice versa, followed by interpolating those mid-points iteratively by cubic spline in a data-driven fashion. For more details about EIA the readers are requested to go through [36]. Moreover, for the ease of understanding the pseudocode of the EIA is given in Table 2. The number of iterations is fixed to 2 based on visual inspection on the full set of data from all the healthy and subjects with knee pathology.

**TABLE 2:** Empirical Iterative Algorithm

Pseudocode of EIA
1. Consider $x(t)$ as an input time series predicated joint angle.
2. For $i = l:2$ where i is number of iterations. a. Identify all local maxima’s as $p^{i}(n)$ and local minima’s as $q^{i}(n)$ of input signal. b. Find mid-point $m^{i}(n) = (x_{m}, y_{m})$ for consecutive maxima $p^{i}(n) = (x_{p}, y_{p})$ and minima $q^{i}(n) =(x_{q}, y_{q})$ and vice versa for all the maxima’s and minimal as: \begin{equation*}\left(x_{m}, y_{m}\right)=\left(\frac{x_{p}+x_{q}}{2}, \frac{y_{p}+y_{q}}{2}\right)\end{equation*} c. Interpolate all the midpoints $m^{i}(n)$ by cubic spline interpolation to get a signal $y^{i}(t)$. d. Consider $y^{i}(t)$ as mput signal: $x(t)$ = $y^{i}(t)$
3. End.
4. Subtract $y^{2}(t)$ from $x(t)$ to get $r(t)$. \begin{equation*}r(t) = x(t) - y^{2}(t)\end{equation*}
5. Where $y^{2}(t)$ is the processed joint angle and $r(t)$ is the residue of the predicted joint angle as noise.

### D. Training

*MyoNet* was trained and tested on Keras 2.2.0 environment configured to use Tensorflow 1.9.0 as backend engine on a 64 bit Ubuntu operating system on Lenovo ThinkStation with an Intel Xeon CPU E5-2650 v2 processor @ 2.6 GHz and 32 GB RAM.

Initially, model is trained for joint angle prediction and later, transfer learning methodology was employed to use the existing features and parameters of feature extractor block for classification of three movements. Thus, enabling sharing of same weights and parameters of feature extractor block for both the classification and prediction provides a cost effective solution for implementation of inference phase.

### E. Performance Evaluation

The performance of the proposed *MyoNet* model for predicting the knee joint angle and classification of movements was measured by different statistical parameters which include mean absolute error, and accuracy, precision, recall, F1-score, confusion matrix respectively. The aforementioned statistical metrics can be expressed by following equations:

\begin{align*} Absolute~Error=&AJ-PJ \tag{2}\\ Precision=&\frac {TP}{TP+FP} \tag{3}\\ Recall=&\frac {TP}{TP+FN} \tag{4}\\ F1score=&2\times \frac {Precision \times Recall}{Precision+Recall} \tag{5}\\ Accuracy=&\frac {TP+TN}{TP+FP+FN+TN}\times 100\tag{6}\end{align*}

where AJ, PJ, TP, TN, FP, and FN represent the actual joint angle, predicted joint angle, true positive, true negative, false positive, and false negative outcomes of the *MyoNet* model respectively.

## IV. Result and Discussion

### A. Prediction of Knee Joint Angle From sEMG Signal

**FIGURE 5.**
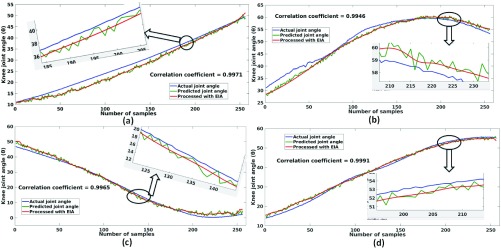
Comparison plots of the actual joint angle, predicted joint angle and EIA processed joint angle for four subjects (a) Healthy subject 3, (b) Healthy subject 7, (c) Subject with knee pathology 1, and (d) Subject with knee pathology 8.

Table 3 depicts the performance analysis of *MyoNet* for prediction of knee-joint-angle. The evaluation is performed using averaged mean absolute error and standard deviation for all the healthy and knee pathology subjects respectively, considering 3 folds validation scheme. The average MAE ± SDAE for all 11 healthy subjects and 11 subjects with knee pathology are 8.1 ± 1.2 and 9.2 ± 1.5 respectively. Fig. 5 shows the comparison plots of actual knee joint angle, predicted joint angle and EIA processed joint angle for healthy subjects 3 and 7, and subject with knee pathology 1 and 8 respectively. The zoomed portion of actual joint angle, predicted joint angle and EIA processed joint angle are also shown in Fig. 5 which are depicted in blue, green and red color respectively. Fig. 5(a), (b), (c) and (d) validate the performance in pictorial form for proposed *MyoNet* model in predicting the knee joint angle.

**TABLE 3:** Performace Evaluation of the Proposed MyoNet for Knee Joint Angle Prediction

Subjects	Healthy	With knee pathology
	MAE± SDAE (%)	MAE± SDAE (%)
1	9.1 ± 0.7	8.8 ± 0.9
2	8.2 ± 1.5	7.3 ± 1.0
3	9.3 ± 0.4	8.4 ± 0.7
4	6.1 ± 2.3	6.4 ± 0.8
5	7.6 ± 1.0	7.6 ± 2.0
6	7.8 ± 1.4	7.2 ± 0.8
7	8.4 ± 1.1	11.4 ± 4.7
8	7.7 ± 1.2	10.3 ± 0.9
9	8.7 ± 1.8	13.1 ± 4.0
10	9.3 ± 0.2	11.4 ± 0.3
11	7.5 ± 2.0	8.6 ± 0.4
Average	8.1 ± 1.2	9.2 ± 1.5

Further, the correlation coefficient of actual joint angle and EIA processed joint angle is estimated which is shown in Fig 5. The correlation coefficient of 0.9971, 0.9946 is obtained for 3^rd^ and 7^th^ healthy subject and 0.9965, 0.9991 for 1^st^ and 8^th^ subjects with knee pathology respectively.

### B. Classification of Three Lower Limb Movements From sEMG Signal

The performance of the proposed *MyoNet* model for classification of the lower limb movements is discussed in this subsection. The *MyoNet* model performance has been quantified and consolidated for all the subjects in Table 4 based on the metrics defined from equations (3) to (5). An average precision, recall and F1-score of 98.8, 97.6, and 98.2 is achieved for all the healthy subjects for movement classification, respectively. Whereas, an average precision, recall and F1-score of 93.4, 92.6, and 92.9 is achieved for subjects with knee pathology, respectively.

**TABLE 4:** Overall MyoNet Model Performance for Healthy and Subjects With Knee Pathology

Subjects	Precision	Recall	F1-score
Healthy	98.8	97.6	98.2
With knee pathology	93.4	92.6	92.9

**FIGURE 6.**
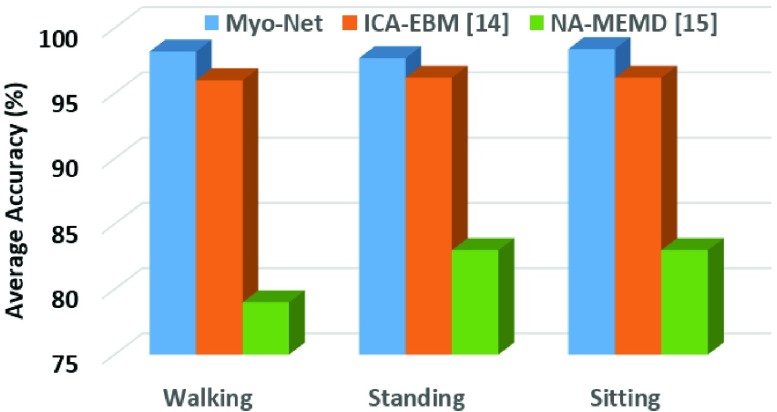
Comparison of proposed MyoNet with ICA-EBM [14] and NA-MEMD [15] for average classification accuracies of walking, standing and sitting for all the healthy subjects.

Automatically, the classification accuracy in percentage of the proposed *MyoNet* model will reveal whether the recorded sEMG signal corresponds to either walking, sitting with knee extension movements or standing with knee flexion movements. Further, we have also listed the subject wise classification accuracy for all the healthy and subjects with knee pathology in Tables 5 and 6 respectively. A comparison of average classification accuracy of proposed *MyoNet* with [14] and [15] for walking, standing, and sitting for all the healthy subjects is illustrated in Fig. 6. The proposed *MyoNet* model achieved an average classification accuracy of 98.2 ± 1.6, 97.7 ± 1.3, and 98.4 ± 1.4 for healthy subjects for walking, standing and sitting respectively. While for subjects with knee pathology, the average classification accuracy of 92.8 ± 1.7, 92.3 ± 1.2, and 98.4 ± 1.4 is achieved for walking, standing, and sitting respectively. Furthermore, the validation of the proposed *MyoNet* model for accurate classification of all the test samples of healthy and subjects with knee pathology for the known classes- walking, standing with knee flexion movements and sitting with knee extension movements are briefly presented in the mean confusion matrix given in Tables 7 and 8 along with a comparison with ICA-EBM [14] state-of-the-art method.

**TABLE 5:** Subject-Wise Classification Accuracy in Percentage for Healthy Subjects for Walking, Standing With Knee Flexion Movements and Sitting With Knee Extension Movements

Healthy Subjects	Proposed MyoNet			ICA-EBM [14]			NA-MEMD [15]		
	Walking	Standing	Sitting	Walking	Standing	Sitting	Walking	Standing	Sitting
1	98.2 ± 1.8	98.5 ± 1.2	97.3 ± 1.8	95.6 ± 1.2	96.3 ± 1.3	96.2 ± 1.3	78.0	76.0	84.0
2	97.6 ± 2.1	97.2 ± 1.6	98.6 ± 1.7	96.3 ± 1.3	96.4 ± 1.3	96.4 ± 1.2	79.0	76.0	84.0
3	97.3 ± 1.7	93.9 ± 1.7	99.2 ± 0.6	95.8 ± 1.4	96.2 ± 1.2	96.1 ± 1.2	83.0	80.0	88.0
4	98.4 ± 0.8	97.3 ± 1.4	99.1 ± 0.2	96.4 ± 1.3	96.3 ± 1.3	96.3 ± 1.1	76.0	76.0	80.0
5	99.1 ± 1.5	99.6 ± 2.2	98.2 ± 2.1	96.1 ± 1.4	95.6 ± 1.1	96.2 ± 1.0	81.0	82.0	76.0
6	98.1 ± 1.6	97.0 ± 1.7	99.5 ± 0.6	95.6 ± 1.6	96.1 ± 1.4	96.3 ± 1.4	84.0	80.0	75.0
7	97.5 ± 2.2	98.5 ± 1.1	98.2 ± 2.1	96.2 ± 1.1	95.8 ± 1.2	96.1 ± 1.1	79.0	89.0	77.0
8	99.4 ± 0.6	95.7 ± 0.7	98.9 ± 1.6	96.3 ± 1.5	96.2 ± 1.3	96.2 ± 1.4	79.0	89.0	86.0
9	96.5 ± 2.6	98.6 ± 1.5	97.3 ± 2.6	95.8 ± 1.3	96.4 ± 1.3	96.1 ± 1.2	79.0	92.0	87.0
10	99.2 ± 1.6	99.8 ± 0.2	98.5 ± 1.6	95.9 ± 1.4	96.5 ± 1.2	96.3 ± 1.2	72.0	89.0	91.0
11	98.8 ± 1.5	99.3 ± 1.3	97.9 ± 1.3	95.8 ± 1.2	96.2 ± 1.5	96.3 ± 1.3	79.0	88.0	86.0
Average	98.2 ± 1.6	97.7 ± 1.3	98.4 ± 1.4	96.0 ± 1.3	96.2 ± 1.2	96.2 ± 1.1	79.0	83.0	83.0

**TABLE 6:** Subject-Wise Classification Accuracy in Percentage for Subjects With Knee Pathology for Walking, Standing With Knee Flexion Movements and Sitting With Knee Extension Movements

Subjects with knee pathology	Proposed MyoNet			ICA-EBM [14]		
	Walking	Standing	Sitting	Walking	Standing	Sitting
1	92.7 ± 1.8	92.1 ± 1.5	90.7 ± 2.1	87.7 ± 1.3	85.8 ± 1.3	86.4 ± 1.4
2	93.6 ± 2.3	91.6 ± 1.6	92.9 ± 1.2	86.8 ± 1.2	85.2 ± 1.2	86.5 ± 1.2
3	94.2 ± 0.7	93.4 ± 0.4	92.6 ± 1.7	86.4 ± 1.3	85.4 ± 1.3	86.7 ± 1.3
4	92.7 ± 2.1	92.9 ± 1.1	91.8 ± 2.2	86.4 ± 1.4	85.5 ± 1.2	86.4 ± 1.2
5	92.6 ± 2.3	91.3 ± 0.7	92.5 ± 1.8	86.8 ± 1.2	85.4 ± 1.4	86.5 ± 1.6
6	92.9 ± 2.4	91.9 ± 0.9	91.4 ± 0.9	86.3 ± 1.3	85.5 ± 1.1	86.4 ± 1.4
7	93.3 ± 1.8	93.6 ± 2.3	92.9 ± 0.7	86.4 ± 1.4	85.7 ± 1.5	86.3 ± 1.3
8	89.6 ± 2.1	92.5 ± 1.2	92.2 ± 1.8	86.3 ± 1.3	85.4 ± 1.2	86.8 ± 1.2
9	94.1 ± 1.0	87.9 ± 1.7	93.1 ± 0.7	86.7 ± 1.2	85.5 ± 1.4	86.3 ± 1.3
10	93.4 ± 0.6	94.8 ± 1.1	92.6 ± 2.2	86.8 ± 1.5	85.8 ± 1.3	86.2 ± 1.4
11	91.9 ± 2.1	93.3 ± 1.3	92.4 ± 2.4	86.4 ± 1.2	85.5 ± 1.4	86.3 ± 1.2
Average	92.8 ± 1.7	92.3 ± 1.2	92.2 ± 1.6	86.6 ± 1.3	85.5 ± 1.3	86.4 ± 1.3

**TABLE 7:** Confusion Matrix for MyoNet Model for Classification Results in Percentage for Healthy Subjects

CLASS	Walking		Standing		Sitting	
	MyoNet	ICA-EBM [14]	MyoNet	ICA-EBM [14]	MyoNet	ICA-EBM [14]
Walking	98.2	96.0	0.8	1.5	1.0	2.4
Standing	1.7	2.3	97.7	96.2	0.6	1.4
Sitting	0.1	1.7	1.5	1.3	98.4	96.2

**TABLE 8:** Confusion Matrix for MyoNet Model for Classification Results in Percentage for Subjects With Knee Pathology

CLASS	Walking		Standing		Sitting	
	MyoNet	ICA-EBM [14]	MyoNet	ICA-EBM [14]	MyoNet	ICA-EBM [14]
Walking	92.8	86.6	5.1	8.6	2.1	7.8
Standing	2.0	5.7	92.3	85.5	5.7	5.8
Sitting	5.2	7.5	2.6	5.9	92.2	86.4

The diagonal bolded element of the confusion matrix represents the success of the *MyoNet* model and state-of-the-art ICA-EBM method [14] in accurately classifying the movements among the known classes while the other elements represent the failure in classifying. Thus, the comparison with [14] and analysis of other parameters such as precision, recall, F1-score and confusion matrix including the classification accuracy shows the statistical significance of the proposed *Myo-Net* model.

### C. Discussion

The lower limb movements including walking, sitting and standing are more likely to be affected in persons suffering from knee related disorders such as knee osteoarthritis, ACL and meniscus injury, restricting them in performing ADL [4]. A recent study showed that sEMG signals captured from the active muscles like quadriceps and hamstring [38] while performing movements, helps the clinician in diagnosis [14], providing assistance in rehabilitation [6] and evaluation of progress in physiotherapy sessions for network based rehabilitation approach [8].

Hence, in viewpoint for remote monitoring of rehabilitation progress, the system demands an efficient, accurate, intelligent and opportune framework, which accurately classify the sEMG signal among the known classes along with the prediction of corresponding knee joint angle information. As detailed in the introductory section, the classification of sEMG signal captured from lower limb muscles suffers from poor accuracy because of complex behavior and cross talk due to overlapping of multiple muscles active at that moment. Many approaches have been listed in the state-of-the-art technologies, among them finding a suitable feature from sEMG signal recording is a common task, which puts extra efforts and induce a delay in assessment. Also, for joint angle prediction sEMG signal were combined with inertial sensor data and FES or goniometers, which put significant amount of battery load in processing and transmitting extra inertial and goniometers data. Further, two different methodologies for movement recognition and joint angle prediction respectively, could prevent generalization of model and may possibly require more energy and processing power for its real-time implementation on wearable devices for envisaged remote monitoring of rehabilitation progress. The deep learning methods emerged as a key factor especially for biomedical applications in providing the accurate performance by carrying out the data-driven feature extraction, obviating the feature selection/extraction steps. Furthermore, the transfer-learning approach helps in the generalization of the model in a single unified platform by utilizing knowledge of the model trained for joint angle prediction to the classification of movements.

For comparison with the state-of-the-art methodologies, a comparative analysis of the proposed *MyoNet* model for movement classification with existing study by [4] and [14-16] is detailed in Table 9 that has used the same dataset included in our study. However, comparison for knee joint angle prediction with the existing study cannot be done because they have used their own data-set for evaluation. The recent study by Naik *et al.* [14], processed the raw sEMG signal with ICA-EBM techniques and then extracted the 7 time-domain features for classification of lower limb movements by LDA classifier. The comparison of person-wise classification accuracy achieved by [14] and proposed MyoNet model for three lower limb movements for healthy and subjects with knee pathology are detailed in Table 5 and Table 6, respectively. Another study by Herrera-Gonzalez *et al.* [4], derived time-frequency and wavelet transform features from sEMG and goniometric signals and utilized multilayer perceptron artificial neural network for the classification of three lower limb movements. Zhang *et al*. [15], considered the decomposed intrinsic mode functions (IMFs) extracted from the raw sEMG signal as features by using EMD, multivariate EMD (MEMD), and noise assisted MEMD (NA-MEMD) for the classification of lower limb movement. However, they had restricted their evaluation to only healthy subjects data, which is detailed in Table 5 for subject-wise comparative analysis. While another study by Ertugrul *et al.* [16], introduced an approach using adaptive local binary patterns (aLBP) for feature extraction from local changes of sEMG signals for binary classification of three lower limb movements. For comparison with state-of-the-art techniques, the average classification accuracy of healthy subjects and subjects with knee pathology is considered and reported in Table 9. The comparison shows the proposed *MyoNet* model outperformed [4], [14], [15] and [16], by 3.9 %, 4.1 %, 13.6 % and 10.2 %. Furthermore, the proposed *MyoNet* model does not involve human intervention in feature selection and extraction unlike the existing methods [4], [14]–[16], while the features are extracted automatically through end-to-end learning from the raw sEMG signals in data-driven fashion. Thus, facilitates high level feature extraction which helps in understanding, determining and distinguishing the hidden information from the sEMG signal regarding their clinical patterns. Thus, it eliminates the need of domain expertise, unlike the state-of-the-art techniques. As a result, enabling a cost-effective solution with enhanced accuracy.

**TABLE 9:** Comparision of Average Classification Accuracy With State-of-the-Art Methodologies

Parameters	[4]	[14]	[15]	[16]	MyoNetmodel
Human intervention in feature selection	Yes	Yes	Yes	Yes	No
Number of different features extracted	39	7	16	6	Data-driven feature extraction
Walking accuracy (%)	88	91.3	79.0	–	95.5
Sitting accuracy (%)	94	91.3	83.0	–	95.0
Standing accuracy (%)	92	90.8	83.0	–	95.3
Average accuracy (%)	91.3	91.1	81.6	85	95.2

This work is a step towards developing an IoT based solution to assist persons suffering from knee related disorders such as knee osteoarthritis, ACL, sciatic nerve injury and meniscus injury in tracking their rehabilitation progress in ambulatory settings by physiotherapist. Further, prediction of joint angle along with movement information can be beneficial for the safety of the rehabilitated subject, which can provide insight if patient is entering into the danger zone and can be informed by sending a vital message. Thus, proposed methodology is helpful in providing proactive assistance to the user and could protect patient from falling and other injuries. In the view of effectiveness of the proposed methodology into remote monitoring of rehabilitation process, we envisaged to develop a wearable device having embedded sEMG electrode integrated with *MyoNet* for proactive decision making and assessment to offer an energy efficient solution by eliminating the energy incurred in data transfer. This output information can be sent to the server accessible by the physiotherapist and patient’s smartphone to interpret the progress remotely. In this study we aimed at detection of lower limb movements along with prediction of corresponding joint angle to track the progress of rehabilitation as a case study. Further, the proposed myoelectric based movement recognition and prediction of corresponding joint angle framework can be suitable for other applications such as prosthetic and exoskeleton control mechanism to assist individuals with amputated limb and paralysis.

## V. Conclusion

A transfer-learning based LRCN deep learning framework named as *MyoNet* is proposed in view of lower limb movement recognition along with prediction of corresponding knee joint angle from sEMG signal recording for remote monitoring rehabilitation progress. The framework is tested on sEMG signal recording from 11 healthy subjects and 11 subjects with knee pathology. This framework precluded handcrafted feature engineering process and enhanced performance by 3.9 %, 4.1 %, 13.6 % and 10.2 % with respect to state-of-the-art methodologies [4], [14], [15] and [16] respectively. The proposed *MyoNet* model was designed considering computational-efficiency and robust architecture by exploiting transfer-learning approach. The *MyoNet* architecture is first of kind where from same sEMG input both classification of movement and prediction of corresponding knee joint angle is done.

In future work we intend to develop a low complex hardware accelerator for the proposed framework for its real-time execution on resource constraint platform.
